# Simultaneous degenerative changes in 2 hepatic cavernous hemangiomas observed over 23 years of follow-up: A case report and review of the literature

**DOI:** 10.1016/j.radcr.2025.09.089

**Published:** 2025-10-29

**Authors:** Toshihiro Kawaguchi, Teruko Arinaga-Hino, Shuichi Tanoue, Tsubasa Tsutsumi, Naofumi Ono, Takumi Kawaguchi

**Affiliations:** a Division of Gastroenterology, Department of Medicine, Kurume University School of Medicine, Kurume, Japan; b Kawaguchi Internal Medicine Clinic, Yanagawa, Japan; c Department of Gastroenterology, Yame General Hospital, Yame, Japan; d Department of Radiology, Kurume University School of Medicine, Kurume, Japan

**Keywords:** Hepatic cavernous hemangioma, Hepatic hemangioma, Hepatic sclerosed hemangioma, Degenerative change, Long-term period

## Abstract

A 54-year-old woman presented to our hospital with general fatigue and liver dysfunction. Following a comprehensive examination, liver dysfunction was attributed to primary biliary cholangitis and autoimmune hepatitis. Abdominal ultrasonography revealed hepatic cavernous hemangiomas measuring 57 and 41 mm, which gradually shrank to 25 mm and 16 mm over 23 years, and the contrast-enhanced computed tomography and magnetic resonance images changed, showing no typical imaging features of hepatic cavernous hemangiomas. These changes were consistent with degenerative processes, such as partial necrosis, fibrosis, and hyalinization, suggesting progression from hepatic cavernous hemangioma to hepatic sclerosed hemangioma. Hepatic cavernous hemangiomas may shrink in size and may change imaging patterns due to degenerative changes over a long-term period.

## Introduction

Hepatic cavernous hemangiomas are the most common non-epithelial tumors, accounting for over 80% of all primary benign liver tumors [1]. Hepatic sclerosed hemangioma is a rare variant of a typical hepatic cavernous hemangioma that develops when a hepatic cavernous hemangioma undergoes degenerative changes such as partial necrosis, fibrosis, and hyalinization [2]. Hepatic sclerosed hemangiomas are benign tumors; however, they can be difficult to differentiate from malignant liver tumors on imaging and may possibly be resected [3]. Seven studies have reported marked degenerative changes with a reduction by more than 1.5cm in the hepatic cavernous hemangiomas [[4], [5], [6], [7], [8], [9], [10]]. However, to our knowledge, there are no reports of degenerative changes in hepatic cavernous hemangiomas with >20 years of follow-up. In addition, no previous reports have described simultaneous changes in multiple hepatic cavernous hemangiomas at different locations. Here, we report a patient with 2 hepatic cavernous hemangiomas demonstrating degenerative changes and a reduction in size over 23 years of follow-up.

## Case report

A 54-year-old Japanese woman presented to our hospital in May 2001, complaining of general fatigue and loss of appetite. No spontaneous pain or tenderness was observed in the abdomen. The liver and spleen were impalpable. Bulbar conjunctivae were not icteric. The palpebral conjunctiva was not anemic. There was no history of alcohol consumption or smoking. The patient had no history of medication use and no family history of liver disease.

Laboratory examination on admission showed elevated levels of hepatobiliary enzymes including aspartate aminotransferase (AST) 449 U/L (normal range: 10-40 U/L), alanine aminotransferase (ALT) 483 U/L (5-40 U/L), alkaline phosphatase (ALP) 267 U/L (80-260 U/L), and gamma-glutamyl transpeptidase (γ-GTP) 133 U/L (< 30 U/L) and total bilirubin 1.3 mg/dL (0.2-1.0 mg/dL). Viral serological tests for viral hepatitis B, C were negative, serum immunoglobulin G (IgG) level was 3110 mg/dL (880-1800 mg/dL), serum IgM level was within normal, anti-nuclear antibody titer was 1:40 (< 40), and anti-mitochondrial antibody was 20 (< 20). Following a liver biopsy, liver dysfunction was diagnosed as primary biliary cholangitis (PBC), and autoimmune hepatitis (AIH) was suspected. Therefore, ursodeoxycholic acid was initiated at a dose of 600 mg/day. Liver function improved rapidly, and liver enzymes returned to normal levels; however, in March 2013+12, the liver function worsened with AST level at 144 U/L (10-40 U/L); ALT, 88 U/L (5-40 U/L); ALP, 332 U/L (115-359 U/L); and, γ-GTP, 44 U/L (< 30 U/L). Serum IgG level was 3118 mg/dL (870-1700 mg/dL), and antinuclear antibody titer was 1:160 (< 40); therefore, we started administering prednisolone at 20 mg/day. Liver dysfunction improved, and the dosage was gradually tapered to a maintenance dose. Based on the above findings, AIH was confirmed with a score of 19 on the revised international diagnostic scoring system for AIH and a score of 7 on the simplified diagnostic criteria, and the diagnosis was PBC–AIH overlap.

**Fig. 1:**
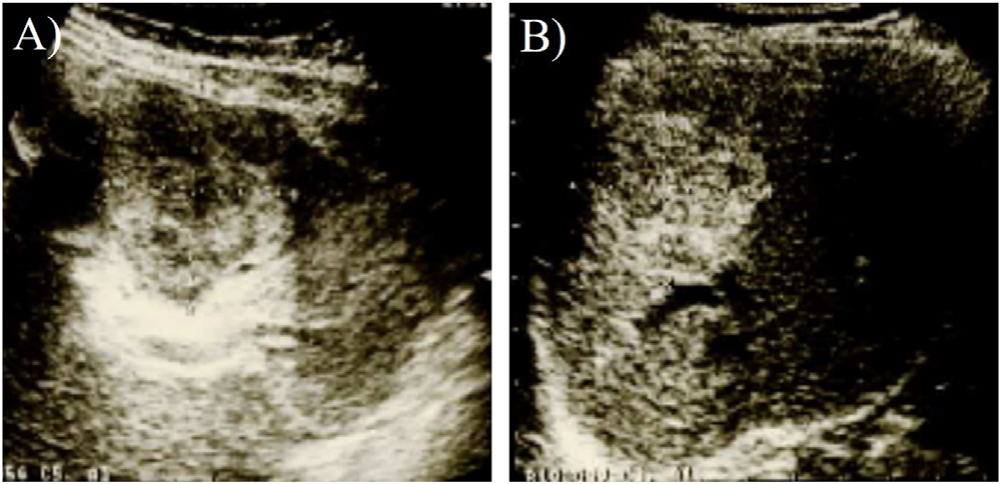
Abdominal ultrasound findings (2001): highly echoic, well-defined lesions measuring 57×51 mm in segment 2/3 of the liver (A) and 41×32 mm in segment 8 of the liver (B) are observed.

Abdominal ultrasound examination performed in 2001 revealed highly echoic, well-defined lesions measuring 57×51 mm in segment 2/3 of the liver and 41 × 32 mm in segment 8 of the liver (Fig. 1). Splenomegaly was not observed. The levels of carcinoembryonic antigen (CEA), carbohydrate antigen 19-9 (CA19-9), α-fetoprotein (AFP), and protein induced by vitamin K absence (PIVKA-Ⅱ) levels were within the normal range. Abdominal post-contrast computed tomography (CT) performed in 2001 showed well-defined masses measuring 55×45 mm in segments 2/3 and 48×35 mm in segment 8 of the liver. Triple-phase contrast-enhanced CT demonstrated peripheral nodular enhancement in the arterial-dominant phase, followed by a progressive centripetal fill-in, suggesting a hepatic cavernous hemangioma. The 2 lesions in the liver at S2/3 and S8 were diagnosed as hepatic cavernous hemangiomas and were monitored using abdominal ultrasonography, CT, and magnetic resonance imaging (MRI).

**Fig. 2:**
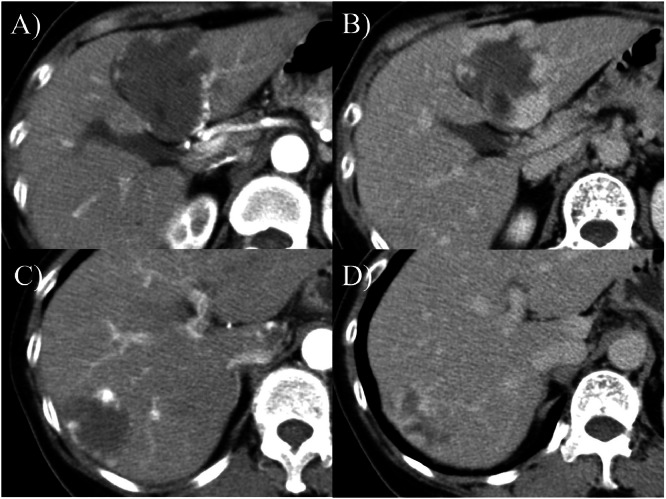
Dynamic abdominal computed tomography findings (2006+5 years): Masses of 58×47 mm in liver S2/3 (A, B) and 34×27 mm in liver S8 (C, D) are observed. In the arterial phase, peripheral nodular enhancement is observed (A, C). In the delayed phase, progressive centripetal filling-in is observed (B, D).

**Fig. 3:**
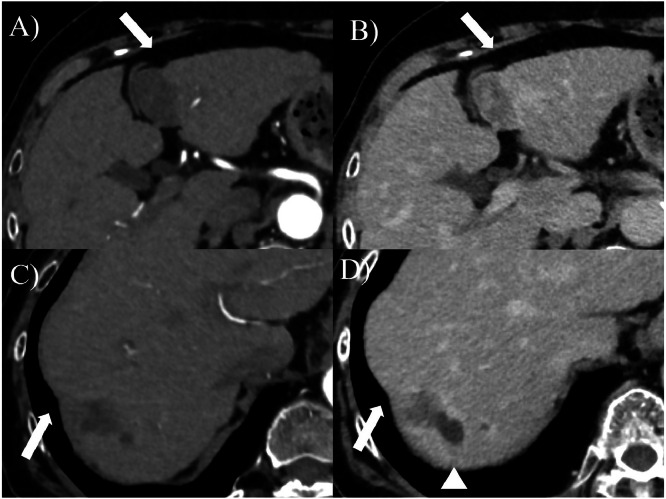
Dynamic abdominal computed tomography findings (2024+23 years). Atrophic masses measuring 29×24 mm in liver S2/3 (A, B) and 18×17 mm in liver S8 (C, D) are observed. In the arterial phase, peripheral nodular enhancement disappeared (A, C). In the delayed phases, only slight contrast enhancement is observed in the center of the tumor (B, D). A concave deformation of the liver surface is observed (arrow). The cyst near S8 has expanded (arrowhead).

**Fig. 4:**
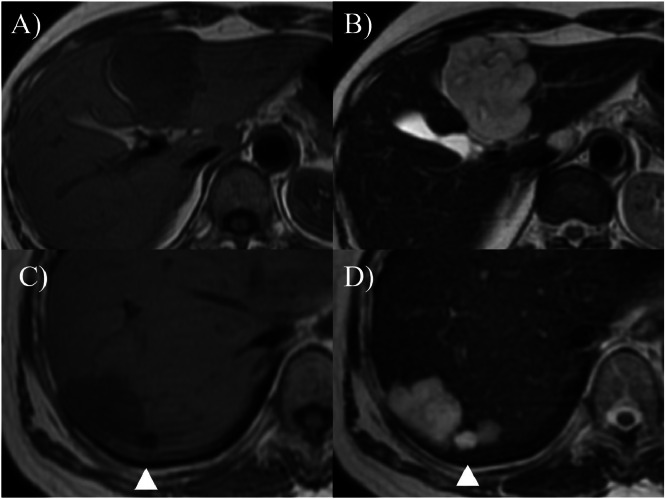
Dynamic abdominal magnetic resonance imaging findings (2007+6 years). A mass of 55×43 mm is observed in liver S2/3 (A, B) and 36×30 mm in liver S8 (C, D). T1WI shows low signal intensity (A, C), and T2WI shows high signal intensity (B, D). A 9 mm cyst is observed near S8 (arrowhead).

**Fig. 5:**
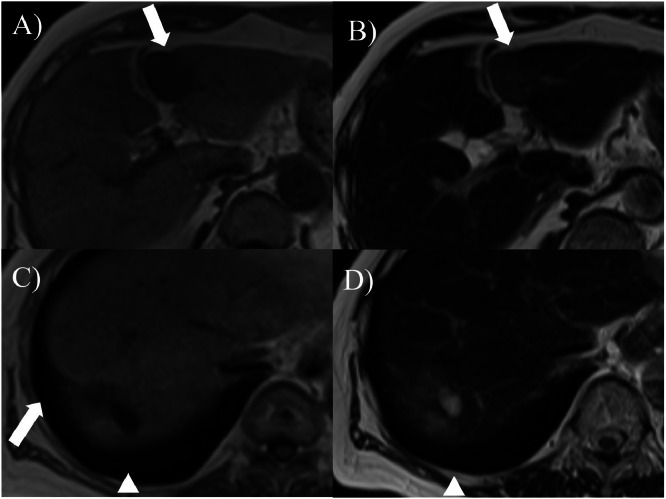
Dynamic abdominal magnetic resonance imaging findings (2001+23 years). Atrophic masses measuring 28×21 mm in liver S2/3 (A, B) and 21×20 mm in liver S8 (C, D) are observed. T1WI shows low signal intensity (A, C), and T2WI shows faint high signal intensity (B, D). A concave deformation of the liver surface is observed (arrow). The cyst near S8 has expanded from 9 to 20 mm. (arrowhead).

The 2 hepatic cavernous hemangiomas gradually shrank over 23 years, each shrinking by more than 2 cm. Abdominal ultrasound examination in 2024+23 showed S2/3 25×24 mm and S8 16×15 mm. The levels of tumor markers, such as carcinoembryonic antigen (CEA), carbohydrate antigen 19-9 (CA19-9), α-fetoprotein (AFP), and protein induced by vitamin K absence (PIVKA-Ⅱ), remained within the normal ranges. Comparative CT and MRI images before and after the follow-up are presented in (Fig. 2, Fig. 3, Fig. 4, Fig. 5). A contrast-enhanced CT scan obtained in 2006+5 demonstrated the characteristic features of hepatic cavernous hemangioma, including peripheral nodular enhancement during the arterial dominant phase and progressive centripetal fill-in in the delayed phase (Fig. 2). By the year 2025+24, follow-up contrast-enhanced CT showed a marked reduction in the size of the lesions: the mass in S2/3 and S8 decreased from 55×45 mm to 29×24 mm and 48×35 mm to 18×17 mm, respectively (Fig. 3). Notably, in both lesions, the peripheral nodular enhancement disappeared in the arterial phase, and only a slight contrast effect was observed in the center of the mass in the delayed phase (Fig. 3). Similarly, the MRI findings revealed interval changes. In 2007+6, contrast-enhanced MRI revealed low signal intensity on T1-weighted images and high signal intensity on T2-weighted images for both lesions (Fig. 4). However, by the year 2024+23, the lesions had decreased in size and exhibited low signal intensity on T1-weighted images and only faint hyperintensity on T2-weighted images (Fig. 5). Peripheral nodular enhancement in the arterial-dominant phase was no longer evident.

**Fig. 6:**
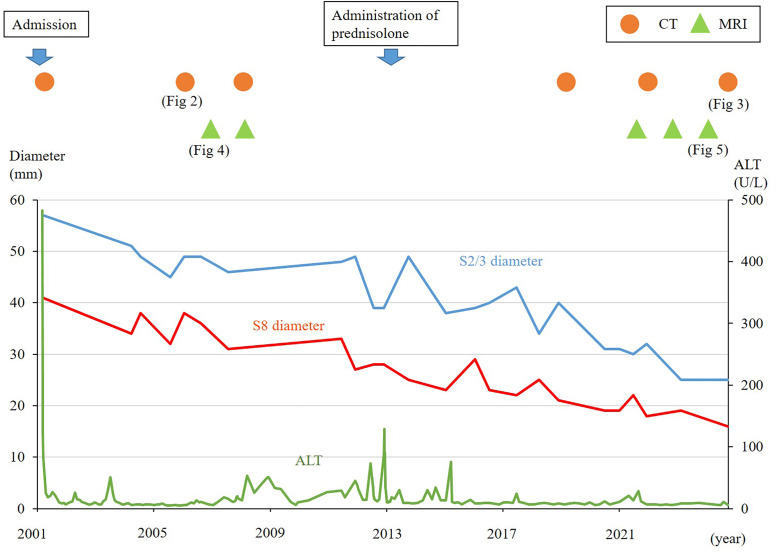
The clinical course of the patient. Changes of tumor diameters measured by ultrasound examination are shown. The 2 hepatic cavernous hemangiomas gradually shrank over 23 years.

These imaging changes were consistent with the degenerative transformation of hepatic cavernous hemangiomas, likely due to fibrosis and hyalinization within the mass, suggesting progression toward hepatic sclerosed hemangiomas. In addition, concave deformities of the liver surface were observed in areas corresponding to the tumor at S 2/3 and S8 (arrows in Fig. 3, Fig. 5). A cyst adjacent to S8 also enlarged from 9 mm to 20 mm (arrowhead in Fig. 3, Fig. 4, Fig. 5). The clinical course of the patient is shown in Fig. 6. The patient is currently under observation.

## Discussion

We observed degenerative transformations in 2 hepatic cavernous hemangiomas over a 23-year follow-up period, with alterations in the imaging patterns observed on contrast-enhanced CT and MRI.

Hepatic cavernous hemangiomas rarely present with degenerative changes such as partial necrosis, fibrosis, and hyalinization, which may result in shrinkage and progression to hepatic sclerosed hemangiomas [2]. In Berry’s autopsy study, such findings were reported in 2 out of every 1000 cases, indicating its relatively rare nature [11].

There have been 7 reports of marked degenerative changes with a reduction by more than 1.5cm in hepatic cavernous hemangiomas (Table 1). However, there are no reports regarding degenerative changes in hepatic cavernous hemangiomas with >20 years of follow-up. Although multiple hepatic cavernous hemangiomas may occur, it is unclear whether they shrink simultaneously. This is the first report regarding degenerative changes in multiples hemangiomas observed simultaneously over 23 years. Among the 8 case reports, including the current case, the male-to-female ratio was 3:5, and the average age was 69 years. The mean tumor sizes before and after degenerative changes were 66 and 31 mm, respectively. The average follow-up period was 11 years, suggesting that such degenerative changes progressed over a long period. Alterations in the imaging patterns on contrast-enhanced CT and/or MRI have been observed in most cases. In 4 out of 8 cases, the diagnosis was pathologically confirmed as hepatic sclerosed hemangioma. The typical imaging features of hepatic cavernous hemangiomas on contrast-enhanced CT include peripheral nodular enhancement in the arterial phase and progressive centripetal filling-in in the delayed phase. On MRI, they show marked hyperintensity on T2-weighted images [12]. In hepatic sclerosed hemangiomas, abundant vascular spaces within the hemangiomas are markedly reduced or lost, leading to atypical imaging findings. Although these findings vary depending on the degree of degenerative change, atypical imaging characteristics, such as no enhancement and ring enhancement, are observed in the arterial-dominant phase of contrast-enhanced CT [13]. On MRI, hepatic sclerosed hemangiomas often appear as a weak signal elevation on T2-weighted images when fibrosis is advanced [14]. In our case, the peripheral nodular enhancement during the arterial-dominant phase, which is a typical imaging feature of hepatic cavernous hemangiomas, disappeared. In addition, MRI demonstrated only faint hyperintensities on T2-weighted images. These changes were consistent with degenerative processes, such as fibrosis and hyalinization, raising the possibility of progression from hepatic cavernous hemangioma to hepatic sclerosed hemangioma. Many cases of hepatic sclerosed hemangioma are discovered incidentally, and differentiating them from malignant hepatic tumors can be difficult, probably because the initial typical features of the hepatic cavernous hemangioma are lost, often necessitating histopathological examination after hepatectomy for diagnosis [3].

**Table 1:** Cases of hepatic cavernous hemangiomas in which marked degenerative changes were observed.

Case	Year	Author	Age (year)		Sex	Tumor size before→after (change) (mm)	Location	Follow-up term	Surface concave change	Contrast-enhanced CT Pattern in an early phase	MRI T1/T2	Pathological confirmation of sclerosed hemangioma
			At diagnosis of hemangioma	After degenerative change								
1	2008	Tsumaki [4]	N/A	70s	F	70→40 (30)	S8	17 years	(+)notable	Marginally enhanced	low/faint high	(+)
2	2011	Miyaki [5]	60s	N/A	F	70→30 (40)	S2/3	12 years	(+)notable	N/A	low/faint high	(+)
3	2012	Doyle [6]	72	N/A	F	N/A	S2	21 months	(+) mild	Ring enhancement	N/A	(+)
4	2013	Shimada [7]	53	63	M	30→15(15)	S8	10 years	(−)	Marginally enhanced	low/faint high	(+)
5	2017	Haradome [8]	(−)	75	M	52→27 (25)	S2/3	21 months	(−)	N/A	N/A/low	(−)
6	2018	Nunes [9]	46	52	M	170→80 (90)	S2/3	6 years	(+)notable	N/A	heterogeneous/high	(−)
7	2024	Hirowatari [10]	40s	N/A	F	35→14 (21)	S2/3	15 years	(+) mild	Marginally enhanced	low/high	(−)
Average of 7 studies			59	68		71→34 (37)		9 years				
8	2025	Kawaguchi	54	78	F	57→25 (32) 41→16 (25)	S2/3 S8	23 years	(+)mild (+)mild	No enhancement No enhancement	low/faint high	(−)

N/A, not available.

Surface deformity of the liver was noted in 6 cases, particularly in those where the original hepatic cavernous hemangioma was large, and shrinkage was marked (Table 1). In the present case, concave changes in the liver surface corresponding to the tumors were observed (arrows in Fig. 3, Fig. 5). Similarly, Doyle et al. [6] reported that 7 of 10 pathologically confirmed hepatic sclerosed hemangiomas had surface indentations corresponding to the lesions.

The proposed mechanisms of degenerative changes in hepatic cavernous hemangiomas include slow blood flow within the lesions, which may lead to repeated cycles of thrombus formation and lysis [15]. Additionally, it has been suggested that, in hepatic cavernous hemangiomas exceeding a certain size, thrombus formation within the tumor triggers localized disseminated intravascular coagulation in the hepatic circulation, contributing to degenerative changes [4]. Other possible contributing factors include aging and hormonal changes after menopause [16,17]. Some studies have documented progressive growth of hepatic cavernous hemangiomas, particularly in association with female sex hormone [16,18]. In the present case, the extended 23-year follow-up of the underlying overlapping PBC and AIH passively allowed us to observe the time course of degeneration in the 2 hepatic cavernous hemangiomas. The contributing factors likely include the original size of the hemangiomas, the long 23-year clinical course, the patient’s older age, and hormonal changes.

In this case, the diagnosis was not confirmed pathologically. Although a definitive diagnosis could have been made by tumor biopsy, the lesions were decreasing in size on imaging, and tumor markers levels were not elevated. Therefore, considering the low possibility of a malignant hepatic tumor, the patient continued to be observed. Two other patients were also managed with observation alone [9,10]. In all of these cases, typical features of hepatic cavernous hemangioma were confirmed prior to the onset of degenerative changes. This suggests that, when a lesion is confirmed as a typical hemangioma, observation without aggressive treatment is warranted, even after degenerative changes have occurred [9].

In conclusion, we encountered a case of hepatic cavernous hemangioma with degenerative changes during a 23-year follow-up period. Hepatic cavernous hemangiomas may shrink in size and may change imaging patterns due to degenerative changes over a long-term period.

## Patient consent

Informed written consent was obtained from the patient for the publication of this report and any accompanying images.
